# Confero: an integrated contrast data and gene set platform for computational analysis and biological interpretation of omics data

**DOI:** 10.1186/1471-2164-14-514

**Published:** 2013-07-29

**Authors:** Leandro Hermida, Carine Poussin, Michael B Stadler, Sylvain Gubian, Alain Sewer, Dimos Gaidatzis, Hans-Rudolf Hotz, Florian Martin, Vincenzo Belcastro, Stéphane Cano, Manuel C Peitsch, Julia Hoeng

**Affiliations:** 1 Philip Morris International Research & Development, Quai Jeanrenaud 5, CH-2000 Neuchatel, Switzerland; 2 Friedrich Miescher Institute for Biomedical Research, Maulbeerstrasse 66, CH-4058 Basel, Switzerland; 3 University of Basel, Petersplatz 10, CH-4003 Basel, Switzerland; 4 Swiss Institute of Bioinformatics, Maulbeerstrasse 66, CH-4058 Basel, Switzerland

**Keywords:** Gene expression, Contrast data, Gene set, Gene set enrichment, Omics, Microarray, Next-generation sequencing, Reproducible research system, Knowledge acquisition

## Abstract

**Background:**

High-throughput omics technologies such as microarrays and next-generation sequencing (NGS) have become indispensable tools in biological research. Computational analysis and biological interpretation of omics data can pose significant challenges due to a number of factors, in particular the systems integration required to fully exploit and compare data from different studies and/or technology platforms. In transcriptomics, the identification of differentially expressed genes when studying effect(s) or contrast(s) of interest constitutes the starting point for further downstream computational analysis (e.g. gene over-representation/enrichment analysis, reverse engineering) leading to mechanistic insights. Therefore, it is important to systematically store the full list of genes with their associated statistical analysis results (differential expression, t-statistics, p-value) corresponding to one or more effect(s) or contrast(s) of interest (shortly termed as ” contrast data”) in a comparable manner and extract gene sets in order to efficiently support downstream analyses and further leverage data on a long-term basis. Filling this gap would open new research perspectives for biologists to discover disease-related biomarkers and to support the understanding of molecular mechanisms underlying specific biological perturbation effects (e.g. disease, genetic, environmental, etc.).

**Results:**

To address these challenges, we developed Confero, a contrast data and gene set platform for downstream analysis and biological interpretation of omics data. The Confero software platform provides storage of contrast data in a simple and standard format, data transformation to enable cross-study and platform data comparison, and automatic extraction and storage of gene sets to build new a priori knowledge which is leveraged by integrated and extensible downstream computational analysis tools. Gene Set Enrichment Analysis (GSEA) and Over-Representation Analysis (ORA) are currently integrated as an analysis module as well as additional tools to support biological interpretation. Confero is a standalone system that also integrates with Galaxy, an open-source workflow management and data integration system. To illustrate Confero platform functionality we walk through major aspects of the Confero workflow and results using the Bioconductor estrogen package dataset.

**Conclusion:**

Confero provides a unique and flexible platform to support downstream computational analysis facilitating biological interpretation. The system has been designed in order to provide the researcher with a simple, innovative, and extensible solution to store and exploit analyzed data in a sustainable and reproducible manner thereby accelerating knowledge-driven research. Confero source code is freely available from http://sourceforge.net/projects/confero/.

## Background

The development and application of high-throughput technologies in biological research has presented researchers with unprecedented amounts of omics data. Management, analysis and interpretation of such data still pose significant challenges. A plethora of open-source software solutions (e.g. caArray [1], MARS [2], BASE [3], EMMA [4], MIMAS [5,6], TM4 [7], MADMAX [8], MiMiR [9], ExpressionPlot [10] to name a few) are readily available for storage and management of raw and preprocessed high-throughput datasets and metadata. These solutions provide a data management platform to facilitate the beginning of the experimental data analysis process. Depending on the complexity of experimental designs, statistical analysis of high-throughput data can involve a number of sophisticated techniques and tools. In transcriptomics, the identification of differentially expressed genes when studying effect(s)/contrast(s) of interest constitutes the starting point for further downstream computational analysis (e.g. gene over-representation/enrichment analysis, reverse engineering, network building, etc.) leading to biological interpretation and mechanistic insights. While many research sites use systems to manage raw and processed data they still do not take advantage of a central downstream infrastructure to store and further exploit analyzed data in an integrated way. In this situation, the value of knowledge gained from analyzed data is restricted to the specific study in which these data were generated, whereas this knowledge could be leveraged during analysis of other studies. Even with the arrival of bioinformatics workflow management systems (e.g. Galaxy [11-13], GenePattern [14], Taverna [15]), which facilitate reproducible analyses, these systems by themselves do not provide the functionality necessary to centrally manage and further utilize analyzed data. Currently, no open-source and free software solutions of this kind exist to store, manage and leverage analyzed data and provide an integrated platform for downstream computational analysis, knowledge acquisition and integration leading to new experimental hypothesis generation. Integration of tools and development of such platforms are important to assemble a systems biology computational workflow supporting interpretation of complex biological data [16].

Here we present an innovative and extensible solution to store and exploit analyzed omics data for the purpose of knowledge acquisition and biological interpretation. Confero enables research sites to store and manage analyzed contrast datasets and identifier (ID) lists of interest (e.g. gene lists extracted from research papers, diagnostic gene signatures), automatically compute and store gene sets from these contrast data and ID lists, and analyze data to support biological interpretation. Confero includes a local database for storage and management of data and metadata as well as tools for downstream computational analysis and biological interpretation, including gene set enrichment analysis (GSEA) and over-representation analysis (ORA) [17]. The Confero Functional Enrichment Analysis module includes specialized tools to facilitate and accelerate enrichment/over-representation analysis and extraction and interpretation of results.

**Figure 1:**
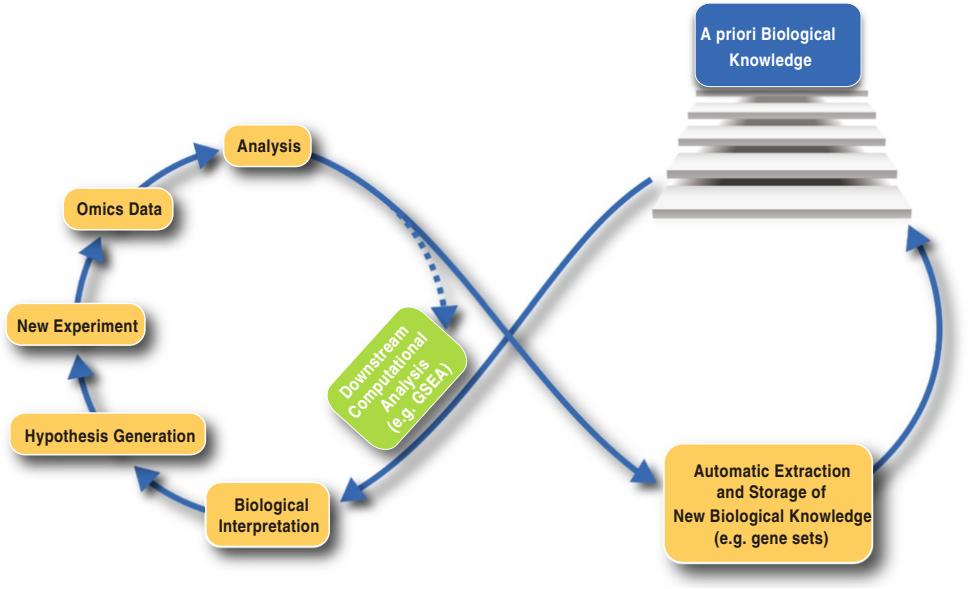
**Biological knowledge building process enabled by Confero which enhances the standard omics analysis workflow.** The left loop depicts the typical omics workflow from data generation, processing, and statistical analysis followed by downstream computational analysis and biological interpretation leading to new hypothesis generation. With Confero in place a second loop is added where statistical analysis results are automatically processed and new *a priori* biological knowledge in the form of gene set is stored. This database of knowledge grows with new experimental and external data and is leveraged by integrated tools for biological interpretation of current and future studies. The overall process drives and enhances the experimental research lifecycle.

The overall goal and spirit of Confero is illustrated in Figure 1. The left loop depicts the typical omics workflow from data generation, processing, and statistical analysis followed by downstream computational analysis and biological interpretation leading to new hypothesis generation. With Confero in place a second loop is added where statistical analysis results are automatically processed and new *a priori* biological knowledge (e.g. gene sets) is stored. This knowledge base grows with new experimental and external data and is leveraged by integrated tools for biological interpretation. The added loop in the analysis workflow facilitates and accelerates knowledge acquisition in biological research, for example in areas such as biomarker and gene function discovery, understanding of molecular mechanisms, and cross-study comparison. The overall process drives and enhances the experimental research lifecycle.

## Implementation

**Figure 2:**
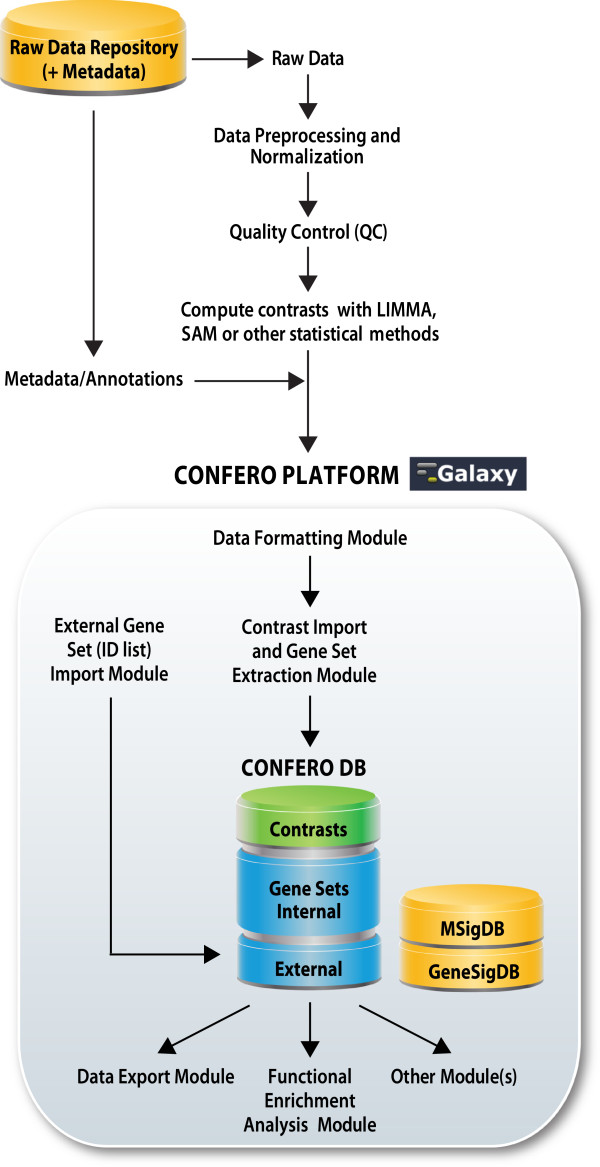
**Confero platform overview.** Depicts where Confero fits into a typical high-throughput transcriptomics analysis workflow. Contrast data is fed into the platform after the statistical analysis step where it is then converted to idMAPS format and loaded using Confero tools. Contrast data is automatically processed and stored and gene sets are extracted. Data can be analyzed for gene set enrichment and results can be used in other Confero tools or exported for other analyses.

As shown in the Figure 2, processing and analysis of omics data involves a number of steps, from data acquisition and transformation, quality control (QC) (e.g. outlier detection, batch effect correction, etc.), preprocessing and normalization, and statistical analysis. According to the experimental design and biological questions of interest, statistical analysis (e.g. pairwise comparisons, multiple linear regression models) is performed to determine the effect(s) of interest (e.g. effect of treatment over time, over dosage, interaction of both time and dose, using pairwise comparison of treated and control samples, etc.) also termed the *contrast(s)* of interest from a dataset.

A contrast corresponds to a quantitative estimate of the differential effect between treatment and reference conditions or more generally as defined by a contrast matrix [18]. Linear models are generally used to estimate the coefficient(s) related to the contrast(s). The estimation of contrast data including differential expression (e.g. most often corresponding to coefficient(s) of the linear model), t-statistics and p-value can be computed for any entity (gene, protein, probe set, transcript, microRNA, etc.) using the Bioconductor [19]*limma*[18] or *samr*[20] packages or other statistical analysis methods [21,22].

**Figure 3:**
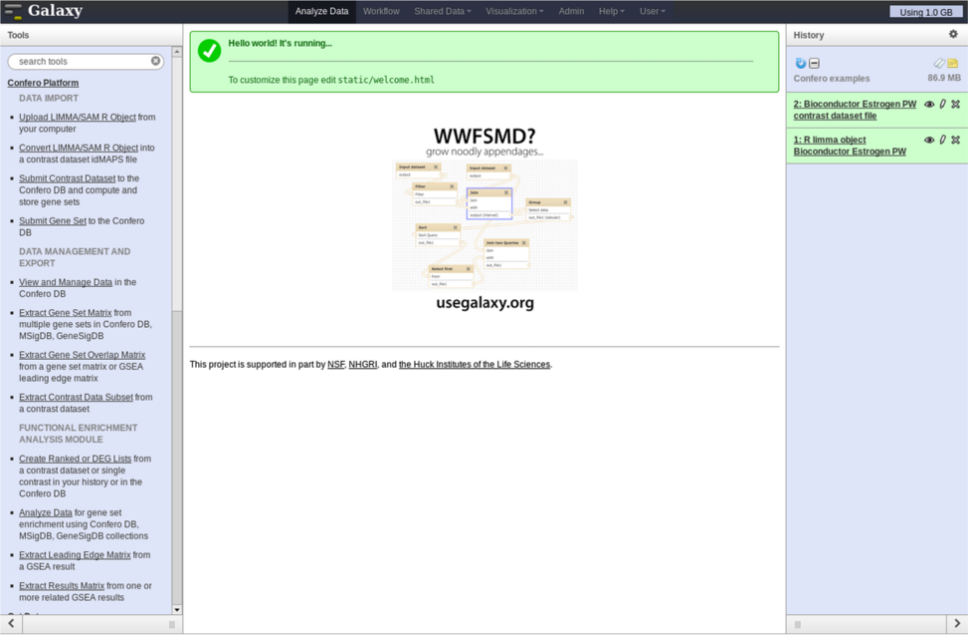
**Screenshot of Confero platform integrated in Galaxy.** Confero platform is a standalone application that can be used via the command line. However, for non-programmatic users and to provide flexibility, Confero platform has been integrated into Galaxy. The window shows three main frames: 1) the first frame on the left contains all Confero tools (also see Additional file 1: Table S1) to import data (DATA IMPORT), manage and export data from Confero (DATA MANAGEMENT AND EXPORT), run GSEA or ORA and manage results (FUNCTIONAL ENRICHMENT ANALYSIS MODULE); 2) the second frame in the middle generally displays the web page with the menu when selecting a tool, and results once the job is done; 3) the third frame on the right contains all the history of actions/results performed during an analysis. This history is saved and the user can investigate the results of the analysis at any time. The user has also the possibility to share the history with other users that have an account in Galaxy.

The Confero platform enables one to 1) convert contrast data coming from statistical analysis into a simple and standard data format, 2) process contrast data and extract gene sets, 3) store contrast data, gene sets and metadata, 4) process and store external ID lists of interest as gene sets, 5) analyze and interpret stored data using integrated tools (e.g. GSEA, ORA) and *a priori* knowledge sources (e.g. Confero DB, MSigDB [17], GeneSigDB [23,24]), and 6) facilitate subsequent downstream analysis with a variety of data transformation and export tools. Confero runs as a standalone system and, as shown in Figure 3, all platform modules are also integrated with the Galaxy workflow management system [11-13]. An overview of all available Confero tools with high-level description is summarized in the Additional file 1: Table S1.

### Advantages and strengths of platform

The Confero platform can serve a variety of different research areas, including biomarker and drug discovery, diagnostics, clinical research, consumer products (e.g. nutrition) or any area that performs omics experiments and analyses. Incorporation of such a platform into a research site’s analysis workflow provides a number of advantages, including that Confero:

•Is open-source, freely installable, customizable, and easily integrates into the Galaxy bioinformatics workflow management system

•Stores, manages, and leverages analyzed omics data

•Enables traceable and reproducible data analysis

•Compiles new biological knowledge (extraction of gene sets from contrast data and population of Confero gene set database) that can be exported and easily shared

•Leverages compiled biological knowledge to analyze (e.g. GSEA or ORA) and support biological interpretation of new contrast data

•Integrates public sources of *a priori* biological knowledge (e.g. MSigDB, GeneSigDB)

•Enables dataset comparison, i.e. systems (*in-vitro* vs. *in-vivo*), organisms (human vs. mouse), treatments (interleukin 1 (IL-1) vs. tumor necrosis factor (TNF)) in a platform-independent manner

•Enables further downstream data mining and meta-analysis of compiled contrast and gene set data, e.g. biomarker discovery, iterative gene set refinement

•Enables incorporation of additional analysis modules (e.g. SAM-GS [25], Running Fisher’s Exact Test [26]) to extend Confero functionalities

### Data formatting

The Confero platform currently supports two types of data input. The first type corresponds to the contrast data resulting from statistical analysis (e.g. microarray or NGS RNA-seq gene expression, microRNA expression, etc.) and the second type can be a simple list of identifiers (e.g. probe set, gene, microRNA, gene symbols, etc.) processed and imported into the Confero database as external gene sets.

### idMAPS file format for contrast data

To support any type of statistical analysis approach, it was necessary to devise a comprehensive yet straightforward file format to represent statistical analysis results. In addition, as Confero requires and leverages various important metadata describing input datasets, it was also necessary that the file format support encoding and passing of such metadata from the upstream workflow in a comprehensive yet independent manner. For this purpose, the idMAPS file format was designed to represent the statistical analysis results of omics experiments including experimental and analysis metadata in fields present in the header of the idMAPS file (e.g. dataset name and description, contrast names, source ID type, etc.). A utility Confero Galaxy tool, *Convert LIMMA/SAMR Object* (R object imported via the *Upload LIMMA/SAM R Object* tool) is provided to convert a Bioconductor *limma* or *samr* R object into an idMAPS file with appropriate header information which can then be used as input for the Confero Galaxy *Submit Contrast Dataset* tool. During the idMAPS file import, metadata are parsed and stored in the Confero database together with contrast data and gene sets. An example of the idMAPS file format is shown in Additional file 2.

### Identifier list file format for external gene sets

In addition to supporting contrast data input, Confero also accepts identifier (ID) lists. An example of an ID list is shown in Additional file 3. This simple file format is a single data column of source IDs with the same Confero metadata file header as in the idMAPS data format.

### Data import, processing and storage

As shown in Figure 3, idMAPS contrast datasets and ID lists are submitted for processing and loading into the Confero database using the Confero Galaxy submission tools *Submit Contrast Dataset* and *Submit Gene Set*, respectively, or via the Confero application programming interface (API). Confero utilizes a comprehensive and robust idMAPS and ID list parser and data integrity checker which, during data processing and submission, will notify users of any problems with their input file.

### Input data identifier (ID) mapping and collapsing methodology

Input idMAPS and ID list data files can use a variety of different source ID types, such as Affymetrix probe set IDs, HUGO gene symbols, and Entrez Gene IDs. To compute gene sets from such data, Confero uses the latest NCBI Entrez Gene [27] annotations to map data to a single gene-centric ID space. For this purpose, a novel and robust ID mapping and collapsing algorithm was developed and includes the following features:

•Source ID-to-multiple Entrez Gene ID mappings are fully supported and handled robustly

•Entrez Gene RefSeq status information is leveraged to determine best mapping genes

•Gene symbol synonyms are supported and properly mapped

•Multiple available collapsing strategies

•Summary report of procedure is generated and stored in Confero database along with each dataset and viewable via the Confero web application

A detailed flowchart describing the Confero ID mapping and collapsing algorithm is shown in Additional file 4: Figure S1.

### Gene set extraction methodology

As prior biological knowledge, a gene set is information commonly utilized to assess enrichment (e.g. GSEA or ORA) of co-regulated genes representative of a specific biological process, pathway, chromosomal location, etc. In the context of contrast data, a gene set corresponds to a set of genes characteristic of an effect of interest. During the Confero submission process, once input data files have completed the ID mapping and collapsing procedure, the Entrez Gene ID-based processed data undergo a novel and robust procedure to extract and store gene sets. The Confero platform builds a gene set database from all imported data that is then leveraged by Confero tools.

Each contrast in a dataset has at least three gene sets automatically generated and named with the following suffixes: the UP (up-regulated genes), DN (down-regulated genes), and AR (all-regulated genes) gene sets. AR gene sets are a special type used to represent the global response of a system to the applied stimulus. The Confero platform provides the user complete and granular control over how each gene set is extracted. As shown in Additional files 2 and 3, special parameters can be provided in the idMAPS metadata header to override default behavior and specify to the algorithm exactly how to proceed. One can also specify to Confero not to create gene sets for a certain contrast (e.g. an intercept coefficient of a linear model) or even for an entire dataset. Different gene set extraction parameters can be specified for each contrast, such as minimum and maximum size thresholds, *P* (significance level, p-value, or false discovery rate (FDR)), *A* (average signal) and *M* (estimated effect of interest, e.g. log_2_ fold change) value thresholds, and even specific desired gene set sizes. A detailed flowchart describing the Confero gene set extraction algorithm is shown in Additional file 5: Figure S2.

### Data management and export

The Confero platform provides an integrated web application to view and manage data and metadata in the Confero database. The web application operates independently of Galaxy but for convenience Confero also embeds it into the Galaxy user interface as the *View and Manage Data* tool. The web application also allows users to export source data, processed data, generated gene sets and data processing reports via the user interface or via the Confero API. Confero also provides an *Extract Gene Set Matrix* Galaxy tool to generate and export boolean gene set-to-gene membership matrices and an *Extract Gene Set Overlap Matrix* tool to extract gene set-to-gene set overlap matrices (i.e. number/percentage of shared genes between two gene sets).

### Functional enrichment analysis module for biological interpretation

Functional enrichment analysis is commonly used to support biological interpretation of gene expression data. The Confero platform currently supports: 1) over-representation analysis (ORA) and 2) gene set enrichment analysis (GSEA), a commonly used and powerful approach for biological data interpretation [17]. An important advantage of GSEA is that full contrast data (e.g. genome-wide expression profiles) can be analyzed in a p-value threshold independent manner unlike other approaches such as ORA.

Both approaches require as inputs a gene list (partial list for ORA and full ranked list for GSEA) and a collection of gene sets used as *a priori* knowledge. A targeted choice of gene sets selected for analyses can provide insight to specific biological questions. We developed a fully integrated Functional Enrichment Analysis module which can seamlessly use Confero contrast data and gene sets (Figure 3; Additional file 1: Table S1). The code and reporting for ORA was developed internally. For GSEA, Confero uses the Broad Institute’s GSEA Java implementation and results reporting [17]. Importantly, Confero dynamically customizes the GSEA results report and provides several tools to accelerate downstream analysis of results. The Functional Enrichment Analysis module includes the following tools:

•*Create Ranked or DEG Lists*: gene lists can be easily generated from contrast data in the Confero database using the statistic (S, moderated t-statistic) or differential expression value (e.g. M, log_2_ fold change) data as the rank metric for GSEA, or using the significance level (P) for ORA (further leveraged to filter the gene list while using the Analyze Data functionality described below).

•*Analyze Data*: ranked and DEG (p-value threshold defined by the user) lists can be analyzed for gene set enrichment/over-representation against dynamically definable Confero gene set collections using annotation filters as well as the latest MSigDB and GeneSigDB gene set collections. The selection of analysis algorithm (GSEA Preranked or ORA (Hypergeometric Test)) conditions the Galaxy menu displayed to choose specific parameters for the analysis.

•*Extract Leading Edge Matrix*: leading edge matrices of various types can be extracted from a GSEA result; the leading edge matrix is comprised of GSEA leading edge genes (in rows) from all gene sets in the result (in columns) passing a specified FDR threshold with rank metric score, rank in list, or boolean membership values as the matrix fields.

•*Extract Results Matrix*: a comprehensive results matrix with user selected output columns can be extracted from one or more functional enrichment analysis results.

In summary, the Confero Functional Enrichment Analysis module allows biologists to compare datasets in a contextual manner (e.g. by organism, cell/tissue type, stimulus type, experimental system, etc.) and to more efficiently identify underlying molecular mechanisms based on biological interpretation of results.

## Results and discussion

### Case study: estrogen bioconductor dataset

To provide an example of the application of the Confero platform, we have used the *estrogen* expression dataset available from the Bioconductor web site [28]. In this 2×2 factorial experiment, MCF7 breast cancer cells were treated with estrogen for 10 or 48 hours. The experimental factors were as follows: “estrogen treatment” with conditions present or absent, and “time” also with two conditions 10 or 48 hours. Following extraction, RNA was hybridized on to Affymetrix HG_U95Av2 microarrays. The purpose of this study was to identify early and late biological processes driven by estrogen putative direct target genes for early response, while for later events the response might be driven by more downstream targets in the molecular pathway.

**Figure 4:**
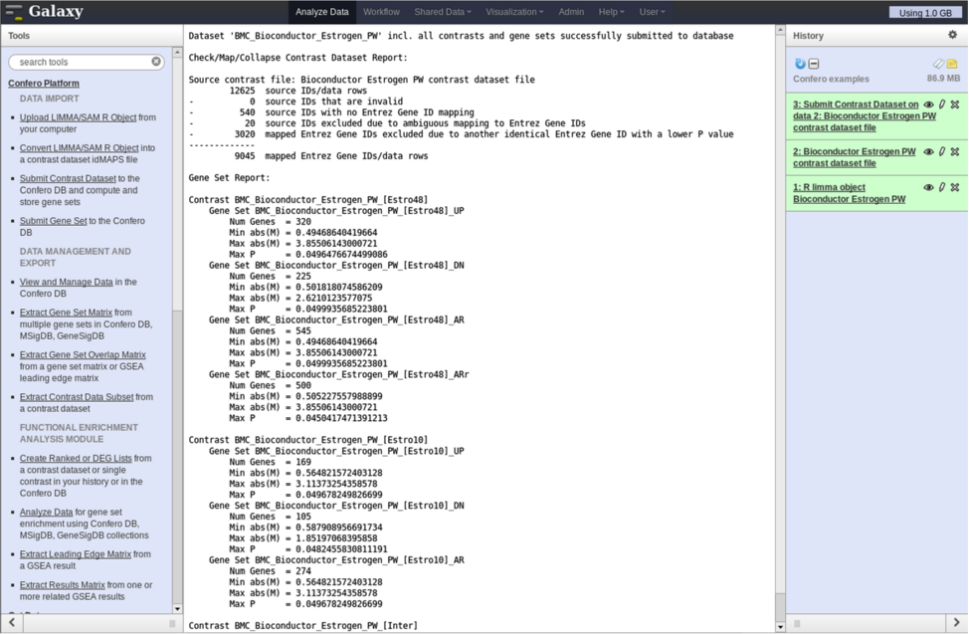
**Screenshot of the report following importing and processing of the Bioconductor *estrogen *contrast data in Confero using Galaxy.** The report shows 1) whether contrast data have been correctly imported and processed (several checks during the mapping and collapsing process reported at the top of the document (“Check/Map/Collapse Contrast Dataset Report”), 2) information on the gene sets that have been automatically extracted from each contrast and stored (UP, DN, and AR gene sets) in the Confero DB (“Gene Set Report”). As example (see also Figure 5), 169, 105 and 274 most significantly up- , down- and all-regulated genes (FDR<0.05) were respectively extracted as gene sets UP, DN and AR from the contrast data “Estro10” (comparison of gene expression levels of estrogen vs control samples collected at 10h).

**Figure 5:**
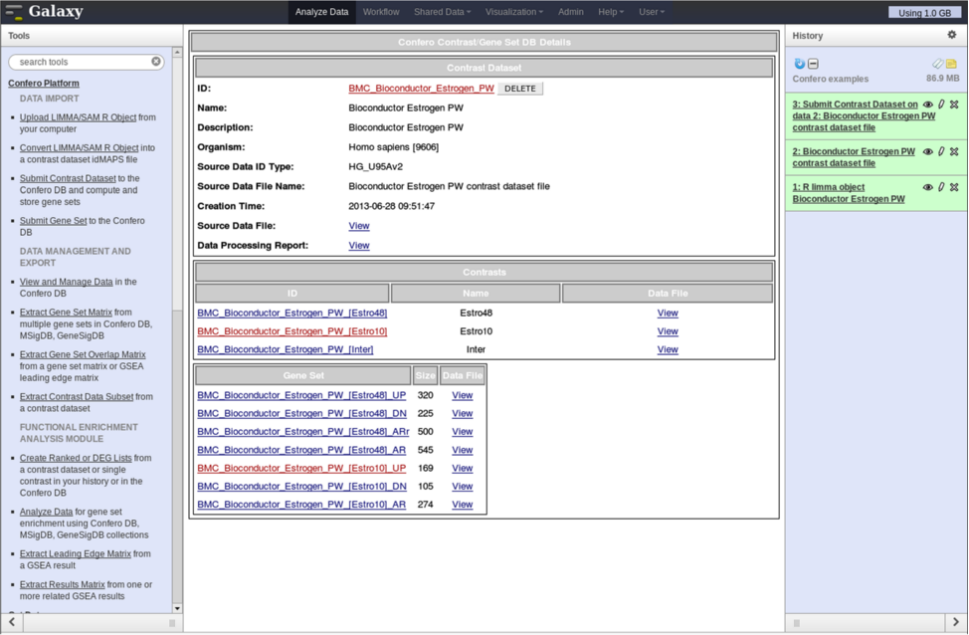
**Screenshot of the Estrogen contrast dataset entry in the Confero DB.** To access a database Estrogen Contrast data entry, the “View and Manage Data” tool is selected in the toolbox (left frame) and the appropriate contrast dataset name can be chosen in the available list. The entry contains information on the contrast dataset at the top (e.g. “BMC_Bioconductor_Estrogen_PW”), contrasts (e.g. “BMC_Bioconductor_Estrogen_PW_[Estro10]”) and associated gene sets at the bottom (e.g. “BMC_Bioconductor_Estrogen_PW_[Estro10]_UP”). Details and data files can be accessed via the hyperlinks. Data files can be saved locally upon needs.

**Figure 6:**
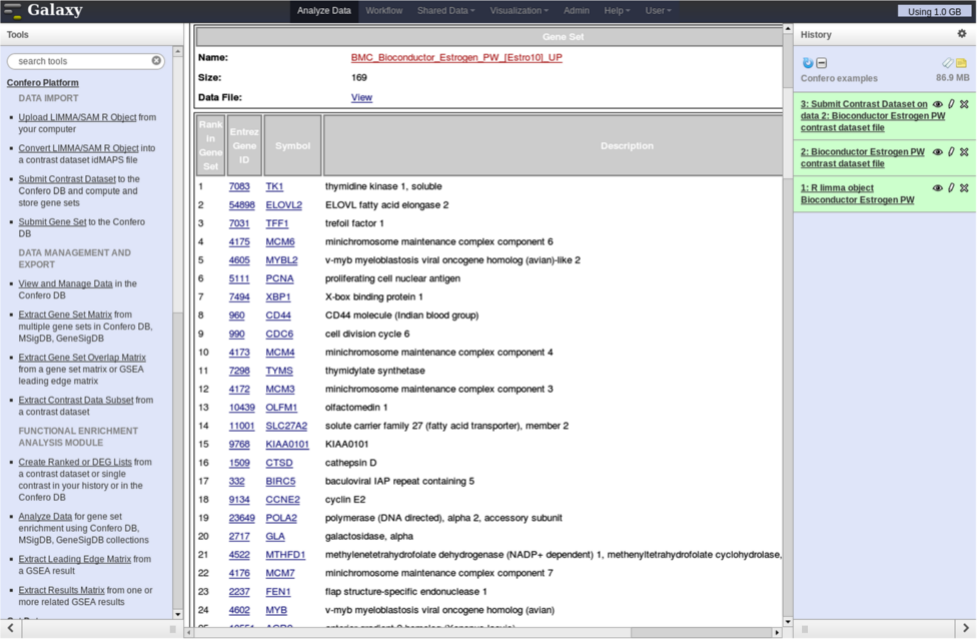
**Screenshot of the Estrogen gene set entry in the Confero DB.** Clicking on a gene set hyperlink (e.g. BMC_Bioconductor_Estrogen_PW_[Estro10]_UP) enables to access detailed information such as the rank of the genes extracted from the contrast, the Entrez gene ID, the gene symbol and description.

Raw data (CEL files) were preprocessed and normalized as described in the Confero platform overview schema (Figure 2). The Bioconductor *limma* package was used to compute contrasts corresponding to the effect of estrogen at 10 (early) and 48 (late) hours (estrogen treatment vs. control comparison for each time point). Additionally, the contrast corresponding to the interaction effect was computed to directly investigate the differential effect of estrogen treatment at 10 and 48 hours. The output *limma* R object from the *eBayes* R function (see Additional file 6) was imported into Galaxy using the Confero *Upload LIMMA/SAM R Object* tool. Data for each contrast, including log_2_ fold changes (M) between control and estrogen treatment conditions, probeset average signal (A), moderated-t statistic (S), and associated FDR (P), were automatically extracted from the R object and converted into idMAPS format using the Confero *Convert LIMMA/SAM R Object* tool (see Figure 3, Additional file 2 and Additional file 1: Table S1). The idMAPS file was then imported into the Confero database using the *Submit Contrast Dataset* tool (Figure 3 and Additional file 1: Table S1). To note, it is also possible to directly import an already formatted idMAPS file into the Confero platform without using the *Convert LIMMA/SAM R Object* tool (Figure 3 and Additional file 1: Table S1). A summary report shows how the contrast dataset was processed and imported as well as information on the gene sets (UP, DN, and AR) extracted and stored for each contrast (Figure 4). In this example, 169, 105 and 274 most significantly up-, down- and all-regulated genes (FDR<0.05) were respectively extracted as gene sets UP, DN and AR from the contrast data “Estro10” (comparison of gene expression levels of estrogen vs control samples collected at 10h). Stored in Confero database, these gene sets represent gene expression perturbation fingerprints of estrogen effect on MCF7 at 10h and could be further leveraged as *a priori* knowledge to analyze and compare new datasets. Contrast data and gene sets derived from the Estrogen data analysis can be accessed and visualized using the *View and Manage Data tool* as shown in Figures 5 and 6.

**Figure 7:**
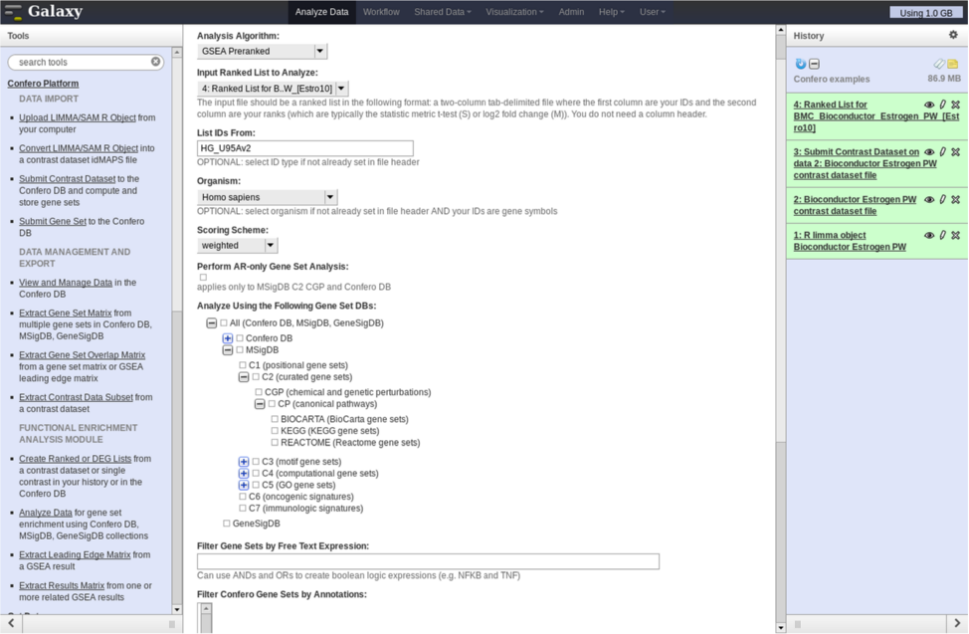
**Screenshot of the Confero Analyze Data Tool (GSEA).** Selecting the analysis algorithm “GSEA Preranked” enables to select specific parameters for GSEA in the Galaxy menu. Confero DB is automatically populated by gene sets as new contrast data or manually curated gene sets are imported into the database. Therefore, having the ability to filter/search gene sets in a specific manner is crucial to have the possibility to address specific biological questions. A filtering/searching functionality (enabling to search gene sets by organism, tissue/cells, stimulus using specific filters or free text expression) is currently available in the *Analyzed Data* tool and will be enhanced in future developments.

**Figure 8:**
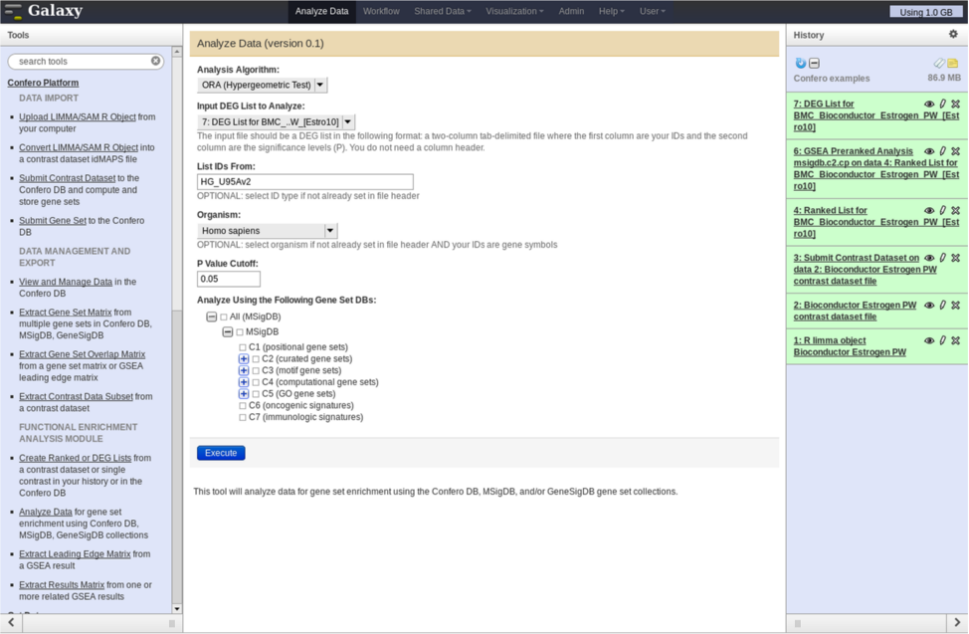
**Screenshot of the Confero Analyze Data Tool (ORA).** Selecting the analysis algorithm “ORA (Hypergeometric Test)” enables to select specific parameters for ORA in the Galaxy menu. MSigDB gene set collections are available for ORA.

**Figure 9:**
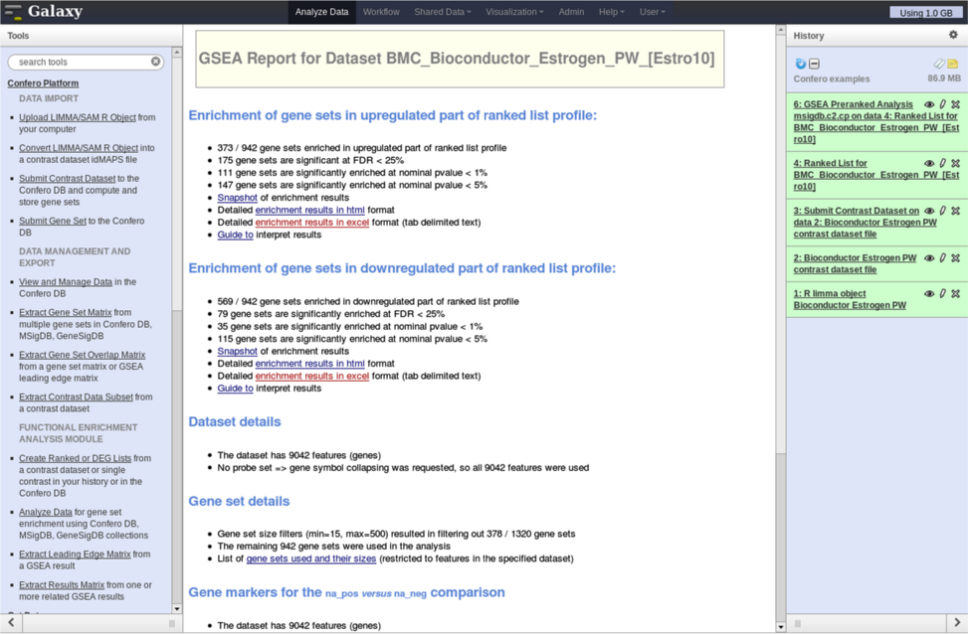
**Screenshot of a GSEA report in Galaxy.** GSEA has been performed with the “BMC_Bioconductor_Estrogen_PW_[Estro10]” contrast using the complete MSigDB C2 gene set collection. The users can navigate in the GSEA report to investigate the results using the hyperlinks of the web page (middle frame).

**Figure 10:**
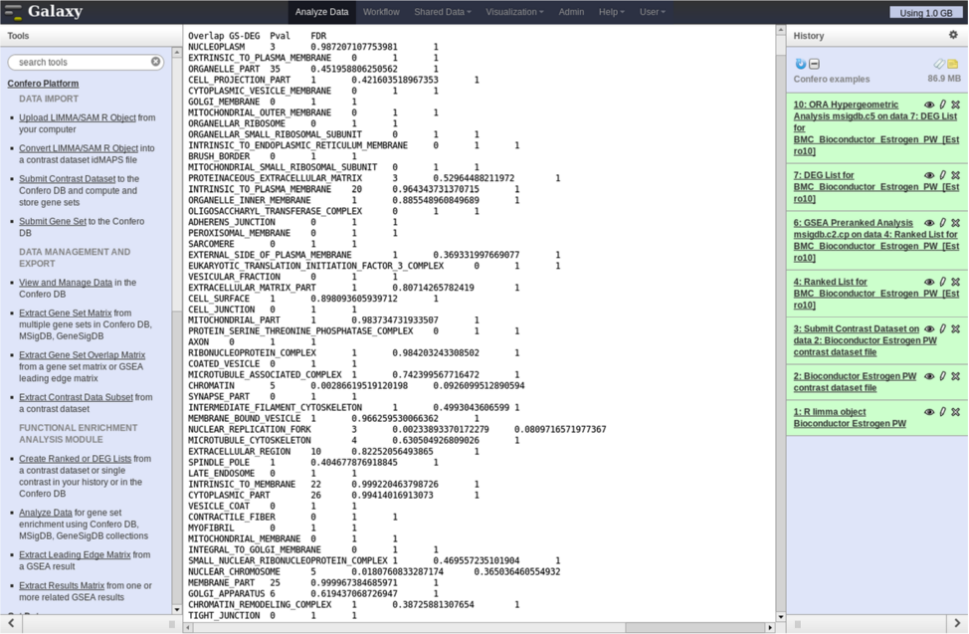
**Screenshot of ORA output in Galaxy.** ORA has been performed with the “BMC_Bioconductor_Estrogen_PW_[Estro10]” contrast using the complete MSigDB C5 gene set collection. The users can visualize the ORA output in Galaxy (middle frame) and easily export the results as a tab-delimited txt file. The file contains 4 columns corresponding to gene set names, number of overlapping genes between the DEG list and gene set, the hypergeometric test p-value and the adjusted p-value (FDR).

To unravel biological processes/pathways regulated by estrogen at the early and late time points, GSEA was performed on the imported contrast data from the *estrogen* dataset. The Confero *Create Ranked or DEG Lists* tool was used to generate a ranked genome-wide differential expression profile for each contrast of the dataset using the moderated-t statistic data column (S) as ranking metric. With these ranked profiles (i.e. “Estro10”, “Estro48”, and “Interaction”) as input, the Confero *Analyze Data* tool was used to set up options and parameters to perform GSEA or ORA (Figures 3, 7 and 8). As an example of GSEA used in the context of this study case, the MSigDB C2 gene set collection from the Broad Institute was selected as *a priori* knowledge. Over time, Confero DB is automatically populated by gene sets as new contrast data or manually curated gene sets are imported into the database. Therefore, having the ability to filter gene sets in a specific manner is crucial to have the possibility to address specific biological questions. A filtering functionality is currently available in the *Analyze Data* tool and will be enhanced in future developments (Figures 3 and 7; Additional file 1: Table S1). The generated GSEA and ORA results for each contrast are directly accessible via the Galaxy user interface (Figures 9 and 10). The investigation of GSEA results through the report on the web is generally a long and tedious process to interpret the results. Indeed, researchers typically have to manually analyze the results sifting through the report for each contrast and drilling down to each gene set to access the associated leading edge genes. Therefore, to facilitate and accelerate analyzing results and biological interpretation, the Confero *Extract Results Matrix* tool was used to extract all related GSEA results into a tab-delimited spreadsheet file (see Additional file 7). The user has the flexibility to select which GSEA results to be extracted. The file contains normalized enrichment scores (NES), NES-associated false discovery rate (FDR) and eventually ranks at which NES is observed in the ranked gene list for all analyzed Estrogen contrast data (i.e. “Estro10”, “Estro48”, and “Interaction”). When interpreting GSEA results, it is generally important to identify which genes contribute the most to the enrichment of significant gene sets. To determine this, the Confero *Extract Leading Edge Matrix* tool was used to extract all leading edge genes from gene sets having FDR values below a user-defined threshold (default value of 0.05) into a single output matrix (see Additional file 8). This customizable output matrix can contain boolean values, moderated-t statistic values (see Additional file 8), or gene rank. This file provides to the biologist more granular molecular insights for interpretation by identifying genes which contribute the most to significant enrichment of observed perturbed biological processes.

**Figure 11:**
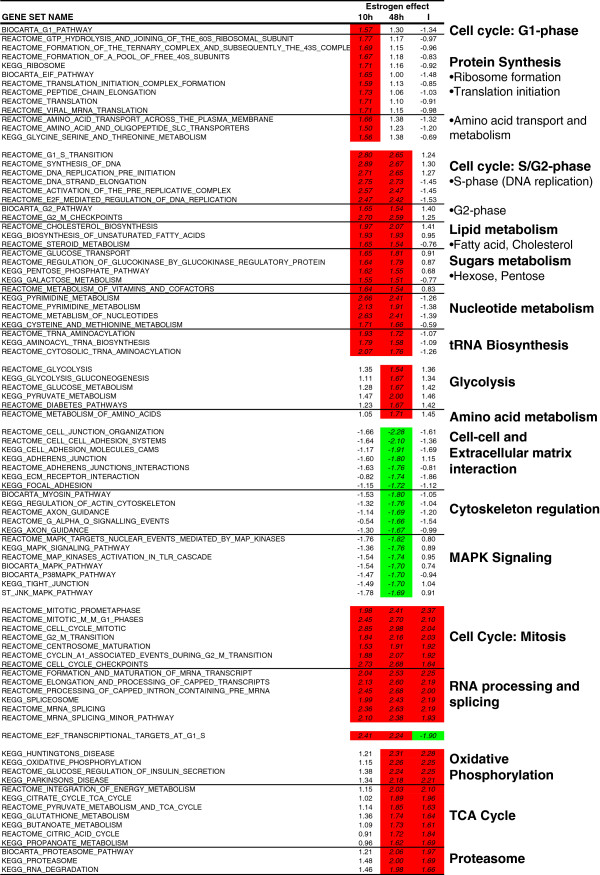
**Summary of Confero Bioconductor *estrogen *dataset GSEA results and leveraging of leading edge genes results from *Export Leading Edge Matrix *tool.** Grouping gene sets per biological processes and investigating the leading edge genes associated to significantly enriched gene sets enables to rapidly interpret biological events at the molecular level and raise new hypothesis that could further be experimentally verified. Red and green colors highlight normalized enrichment scores that are significantly enriched for up- or down-regulated genes, respectively.

Overall, the result files generated by the Confero platform tools enable more rapid and efficient biological interpretation of data. Indeed, the GSEA results matrix can be directly leveraged to identify significant gene sets per contrast and also search for enrichment patterns across contrasts similarly to Figure 11. Grouping gene sets per biological processes and investigating the leading edge genes associated to significantly enriched gene sets enables to rapidly interpret biological events at the molecular level and raise new hypothesis that could further be experimentally verified. As shown in 11, the results highlight that processes corresponding mainly to cell cycle and metabolism were activated in MCF7 cells exposed to estrogen. The pattern of gene set enrichment over time seemed to indicate that the proportion of MCF7 cells in different phases of the cell cycle diverged at early and late time points. Indeed, enrichment of genes representative of the G1 and S-phases were more important at 10 hours, whereas enrichment of genes involved in G2 and M-phases was predominant at 48 hours. Therefore, it was possible to follow the enrichment profile over time for genes implicated in processes coupled to growth and division of cells: activation of protein synthesis machinery, lipid and sugar metabolism to provide energy to the cell, nucleotide metabolism required for DNA replication, amino acid metabolism for protein synthesis, and decrease of cell-cell and extracellular interaction as well as cytoskeleton function, which is a phenomenon characteristic of cells under proliferation. Similar observations have been reported by other studies investigating gene expression profiles of MCF7 exposed to estradiol [29,30]. Only at the later time point (48 hours), genes involved in oxidative phosphorylation and TCA cycle were highly enriched. This observation might suggest that either cell mitosis is accompanied by mitochondria biogenesis [31], or that estrogen regulates the transcription of those genes. Independent studies seem to support the latter hypothesis. Indeed, estradiol has been shown to enhance the transcript levels of mitochondrial genome-encoded genes in several cell types such as MCF7 [32,33]. In hepatocytes, this effect was accompanied by an increase of the mitochondrial respiratory chain activity [33].

### Galaxy integration and workflows

The Confero platform functions as a standalone system and, as shown in Figure 3, can also be fully integrated into the Galaxy reproducible research framework which allows sites to easily incorporate Confero into their existing analysis and knowledge acquisition workflows. Galaxy offers the possibility to chain as well as parallelize, in a customized and flexible way, Confero tools via Galaxy’s workflow framework. For example, the Confero Functional Enrichment Analysis module tools can be connected in a workflow to efficiently analyze data and extract results in parallel.

### Public data update and confero database reprocessing

Vendor technology platform and public Entrez Gene information and annotations update frequently and since all Confero gene sets are computed utilizing this information over time the Confero gene set database would become stale and out-of-date. In addition, if a site chose to change Confero platform configuration parameters for data processing and/or gene set extraction it would be important that this change propagate not only to new data but to all existing data stored in the Confero database. An important and powerful management feature of the Confero software platform is the ability to automatically download and update all relevant and supported vendor technology platform and public gene information and then, using this information, fully reprocess all data contained in the Confero database utilizing current desired configuration parameters. Processed datasets are tagged with processing date and annotation build versions to ensure analysis reproducibility. This functionality is provided by a management program within the Confero distribution.

The Confero distribution currently supports Affymetrix, Illumina, and any NCBI GEO-derived microarray platform as well as all HUGO gene symbols. The management program automatically downloads source annotation files and Entrez Gene information to generate new mapping files. Adding support for other technology platforms (e.g. Exiqon, Nimblegen, etc.) is also possible. For NGS platforms, currently gene level data and analysis is supported.

### Management features and application programming interface (API)

Confero provides a number of useful management functionalities neatly packaged as server-side programs for administrators and software platform managers. This includes programs to perform the following tasks:

•Process and submit batches of contrast datasets or ID lists

•Download, process, and load the latest vendor annotations and Entrez Gene information to generate new Confero source-to-Entrez Gene ID mapping files and fully reprocessing all Confero database data

•Control the Confero embedded web server and application

•Export the entire Confero gene set database with annotations

The API currently provides functions to export different data from the platform.

### Comparison to existing software

The Confero platform provides a unique set of functionalities and differs from existing publicly available software in a number of key aspects. Currently, there are other systems, such as Cistrome [34] and the Genomic HyperBrowser [35], which also have Galaxy integration. In contrast with Confero these systems are focused on certain types of omics data types and are not provided as a software distribution that can be installed locally to work with private as well as public data. Other systems such as TM4 [7,36], provide some similar analysis functionalities such as GSEA. Yet these systems are only all-in-one standalone solutions, not integrated with popular workflow management systems like Galaxy, and do not process imported data to build a database of *a priori* biological knowledge that is then leveraged by system tools. Finally, ArrayExpress and GEO, the two major public repositories, provide tools [37-41] to mine data across a subset of curated and preprocessed studies in their databases. However, such tools are clearly limited to what is available in their systems and cannot be locally installed to work with private data. In addition, many of these systems start with raw/preprocessed data and perform automated statistical analysis, which in Confero a deliberate design choice was made to allow each site the freedom to have customized statistical analysis approaches appropriate for each experimental design and relevant biological questions.

## Conclusions

Storage and exploitation of analyzed omics data is a crucial component of a research site’s analysis workflow as it enables acquisition of new biological knowledge which facilitates interpretation of data. However, these needs are not met by the current open-source solutions freely available to researchers. The Confero platform has been built to provide an innovative, flexible and extensible solution to store and leverage analyzed data and build new *a priori* biological knowledge. In addition, Confero enables cross-platform comparison of omics data in a traceable and reproducible manner.

While GSEA or ORA provide a useful global overview of the perturbed biological processes occurring during an experiment, there are a number of potential methods that can further utilize Confero data to gain a more detailed understanding of underlying mechanisms. We are currently developing additional integrated tools in the following areas:

•Clustering module to group Confero data (e.g. gene sets, genes, etc.) in order to highlight patterns of co-regulation in experimental data

•Visualization module to provide integrated and easy-to-use plotting functions (e.g. volcano plots) on Confero results

•HomoloGene infrastructure and integration to provide cleaner species-to-species translation during gene set enrichment analysis

•Incorporation of additional analysis tools to provide complementary approaches to GSEA for biological interpretation

## Availability and requirements

An overview of the Confero platform software architecture is shown in Additional file 9: Figure S3. Confero is written using the Perl [42] programming language and requires Perl 5.12 or higher. To use the *Convert LIMMA/SAMR Object* tool, an R version and the Bioconductor *limma* and *samr* packages should be installed. The Confero platform requires a backend database and MySQL [43] is currently supported. MySQL 5.0 or higher (5.1 and 5.5) have been fully tested and are being used in production installations. Confero can be installed on any UNIX like operating system (e.g. BSD, Linux, etc.) and has been fully tested and used in production using the Linux operating system. The documentation for the Confero command line interface (CLI) is provided as Additional file 10 and is also available on SourceForge (http://sourceforge.net/projects/confero/).

The Confero distribution comes with an interactive setup program to guide administrators through installation and configuration of the entire software platform for a site. This includes automatic download and installation of dependencies, creation and initial population of the database, Galaxy server integration, and automatic download, processing and loading of all required reference data to begin using the platform. For Confero terms of use and installation, you will have to refer to the DISCLAIMER and INSTALL files provided with Confero software freely available on http://sourceforge.net/projects/confero/ and released under the GPLv2 license.

## Abbreviations

API: Application programming interface; CPAN: Comprehensive perl archive network; DBMS: Database management system; FDR: False discovery rate; GEO: Gene expression omnibus; GO: Gene ontology; GSEA: Gene set enrichment analysis; ORA: Over-representation analysis; DEG: Differentially expressed genes; NGS: Next-generation sequencing; QC: Quality control; CLI: Command line interface.

## Competing interests

The authors declare that they have no competing interests. PMI and FMI authors performed this work under a research collaboration funded by PMI.

## Authors’ contributions

LH, CP, MBS, AS, DG, and HRH conceived the project and developed algorithms and feature requirements. LH designed and wrote all software and database platform code except for LIMMA/SAMR-to-idMAPS converter tool code designed and written by SG. The ORA tool has been designed, written and integrated by CP, SG and SC. FM and VB made significant contributions to the mapping and collapsing algorithm. SG and SC have released Confero software on Sourceforge. LH and CP wrote the manuscript. JH and MCP contributed to the manuscript and supported the project. All authors read and approved the final manuscript.

## Supplementary Material

Additional file 1: Table S1 Overview of tools available in Confero. Click here for file

Additional file 2 **Example of idMAPS file format from Bioconductor *estrogen *dataset.** The file format includes the following characteristics: • Simple, tab-delimited file format with data column header row and file header to encode metadata and other important user-defined parameters relevant to data processing. • The first data column always contains the source IDs for each data row. Source IDs can come from any technology platform supported by Confero, i.e. any platform where ID mapping data is defined. • Data column header letters represent the important statistical analysis results: *M* is the estimated effect of interest (e.g. log_2_ fold change), *A* is the average signal, *P* is the significance level (p-value or false discovery rate (FDR)), and *S* is the statistic (e.g. moderated t-statistic). • Contrast data derived from the same statistical analysis (i.e. a contrast dataset) are represented by repeating groups of data columns (e.g. MAPSMAPSMAPS…) as a matrix in the same file. • Data columns may be in any order (e.g. APSM, SAPM, MASP, etc.) as long as they are in the same order for each contrast (e.g. AMSPAMSPAMSP…). • Certain data columns may be omitted if they do not exist. The minimum requirements are the data column *M* and that each group within a dataset must have the same data column(s). Additional arbitrary data columns not used by Confero may be included for each contrast which are stored and passed through during data processing. Click here for file

Additional file 3 **Example of ID list file format using Gene Ontology (GO) Inflammatory Response biological process.** The file contains header lines that provides a description of the gene list (“gene_set_desc”), indicates the type of ID used (Entrez gene ID can also be used) (“id_type”) and the organism from which the gene list is derived from. Click here for file

Additional file 4: Figure S1 Confero dataset ID mapping and collapsing algorithm flowchart. This figure depicts the steps enabling to go from the original idMAPS data matrix to a mapped and collapsed data matrix ready for downstream GSEA or other analysis which requires no multiple probesets per gene (Gene centric). Click here for file

Additional file 5: Figure S2 Confero gene set extraction algorithm flowchart. The figure depicts the steps to extract up- (UP), down- (DN) and all- (AR) regulated genes from mapped and collapsed contrast data leading to the creation of new gene sets stored in Confero DB. Click here for file

Additional file 6 **R *limma *object from Bioconductor *estrogen *dataset pairwise comparison statistical analysis.** Click here for file

Additional file 7 **Bioconductor *estrogen *dataset MSigDB canonical pathways GSEA results matrix from *Extract Results Matrix *tool.** All GSEA results for the analysis of several contrasts can be extracted in a single file using the *Extract Results Matrix* tool.The columns respectively correspond to gene set name, normalized enrichment scores (NES), NES-associated false discovery rate (FDR) and rank (for which the maximum enrichment score is identified in the ranked gene list) for the selected analyzed contrasts. The tool provides flexibility to select the contrasts and GSEA result parameters to be exported. Click here for file

Additional file 8 **Bioconductor *estrogen *dataset 10 hour time point MSigDB canonical pathways GSEA leading edge matrix from Confero *Extract Leading Edge Matrix *tool.** When interpreting GSEA results, it is generally important to identify which genes contribute the most to the enrichment of significant gene sets. The Confero *Extract Leading Edge Matrix* tool was designed to extract all leading edge genes (as rows) from gene sets (as columns) having FDR values below a user-defined threshold (default value of 0.05) into a single output matrix. This customizable output matrix can contain boolean values, moderated-t statistic values (current view), or gene rank. Click here for file

Additional file 9 **Confero software architecture schematic diagram showing the various platform components and how they integrate with each other.** Users currently interact with Confero via the web browser or command line interface (CLI). Users execute Confero tools via the Galaxy user interface or CLI commands and can view and manage data in Confero via the Confero web application. Users can leverage Galaxy functionalities with Confero tools such as workflow building and execution, parallel and asynchronous job processing, and sharing and publishing of results. Confero administration is performed using management scripts which update annotations from NCBI Entrez Gene, Affymetrix, Illumina, Agilent, and GEO and then can reprocess Confero DB data using updated annotations. Click here for file

Additional file 10 **Confero command line interface documentation including examples.** Confero can be used via Galaxy web interface or command line interface for a programmatic usage of the platform. Click here for file
