# Supplementary material for: Confero: an integrated contrast data and gene set platform for computational analysis and biological interpretation of omics data
Source: BMC Genomics. 2013 Jul 29;14:514. doi: 10.1186/1471-2164-14-514 (PMC3750322; doi:10.1186/1471-2164-14-514)
Supplement: Additional file 1: Table S1 — Overview of tools available in Confero. [file 1471-2164-14-514-S1.docx]

Supplementary Table 1: Overview of tools available in Confero

| **MODULE** | **TOOL NAME** | **PURPOSE** |
| --- | --- | --- |
| Data Import | Upload LIMMA/SAM R Object | Tool to upload a limma or sam R object in Galaxy. |
|  | Convert LIMMA/SAM R object | Tool that converts the uploaded R objects into the standard idMAPS file format. |
|  | Submit Contrast Dataset | Tool to submit the contrast dataset as idMAPS format to Confero DB (1). |
|  | Submit Gene Set | Tool to submit gene list as idMAPS format in Confero DB (1). |
| Data Management and Export | View and Manage Data | Tool to view and manage contrast and gene set data present in Confero DB. |
|  | Extract Gene Set Matrix | Tool to extract a “Gene Set” matrix with columns corresponding to selected gene sets and rows to genes (0 = gene not present in the gene set; 1 = gene present in the gene set). |
|  | Extract Gene Set Overlap Matrix | Tool to extract squared matrix containing the number or percentage of genes which overlap between selected gene sets from a gene set or GSEA leading edge matrix. |
|  | Extract Contrast Data Subset | Tool to extract contrast data subset from a whole contrast dataset. |
| Functional Enrichment Analysis Module | Create Ranked or DEG Lists | Tool to sort the list of genes by t-statistics or log_2_ fold change to be used by GSEA. Tool to create gene list with p-values for further selecting differentially expressed genes (DEG) for over-representation analysis (ORA). |
|  | Analyze Data | Tool to set up GSEA or ORA parameters, select gene set collections or specific gene sets (searching functionality), and run GSEA Preranked algorithm. |
|  | Extract Leading Edge Matrix | Tool to extract from GSEA result files leading edge genes (genes contributing to the maximum enrichment score) for significantly enriched gene sets (threshold to set up). Same format as gene set matrix (0 = 0 gene absent; 1 = gene present). |
|  | Extract Results Matrix | Tool to extract and combine GSEA results of several contrasts in a single file. User can select the parameter results to be exported. |

1. The system automatically processes the imported data using the *Input Data Identifier (ID) Mapping and Collapsing methodology* (Supplemental Figure 1), and extract gene sets from contrast data using the *Gene Set Extraction methodology* (Supplemental Figure 2).
