# Supplementary material for: Confero: an integrated contrast data and gene set platform for computational analysis and biological interpretation of omics data
Source: BMC Genomics. 2013 Jul 29;14:514. doi: 10.1186/1471-2164-14-514 (PMC3750322; doi:10.1186/1471-2164-14-514)

Technology Platform Source-to-Entrez Gene ID Mapping (Preprocessing)

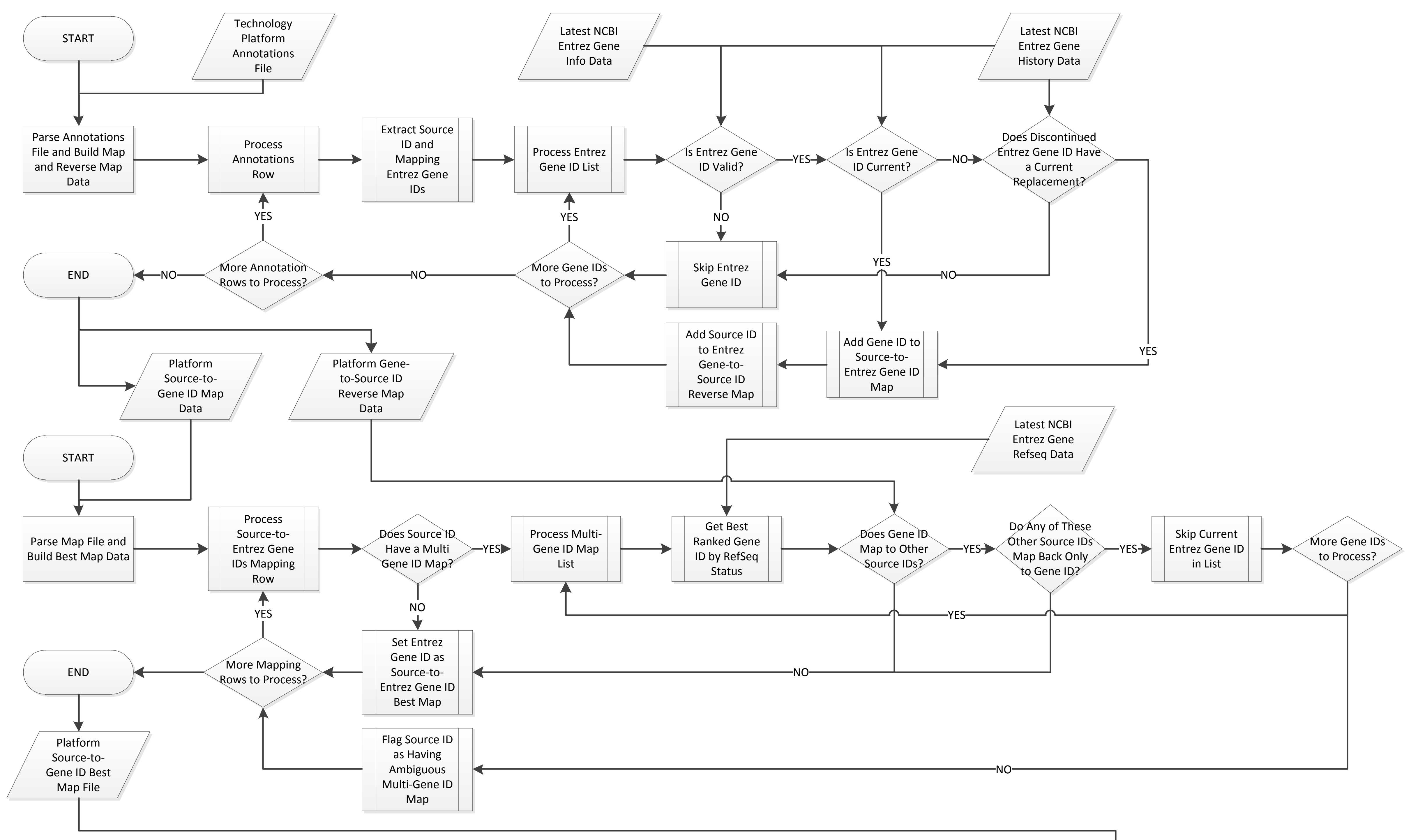

HUGO Gene Symbol-to Entrez Gene ID Mapping (Preprocessing)

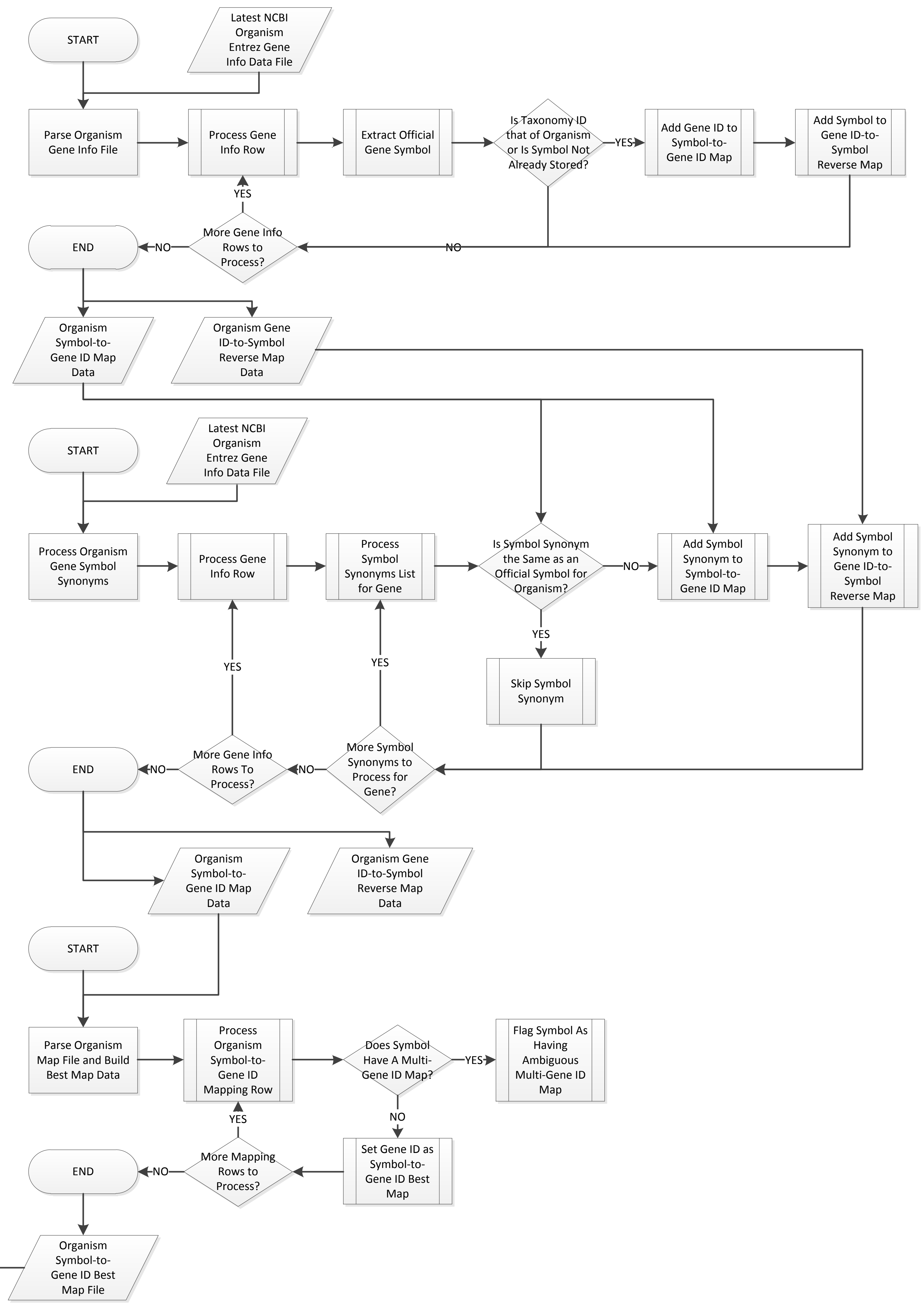

Source or Gene Symbol-to-Entrez Gene ID Mapping and Collapsing (Runtime)

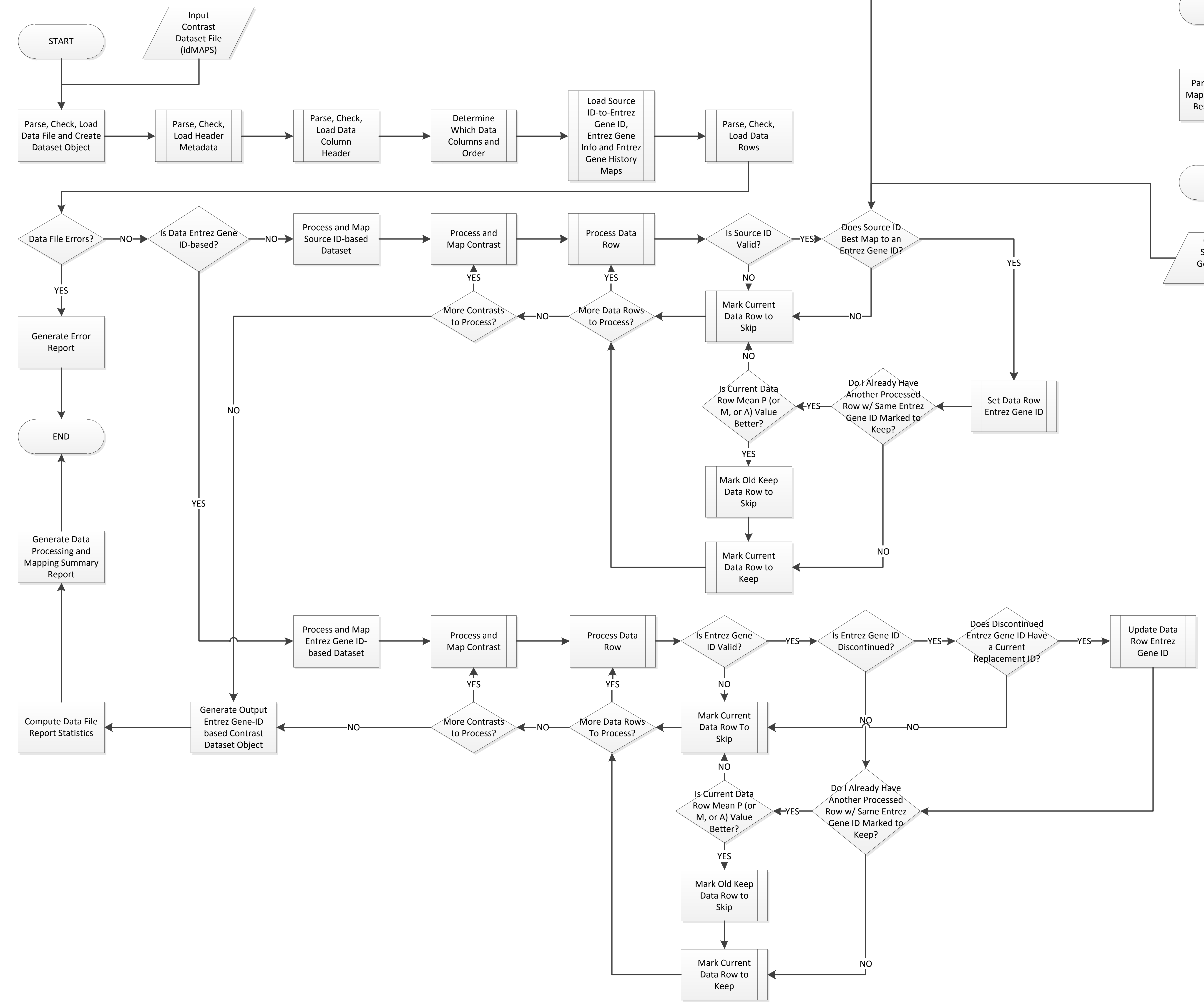

Supplement: Additional file 4: Figure S1 — Confero dataset ID mapping and collapsing algorithm flowchart. This figure depicts the steps enabling to go from the original idMAPS data matrix to a mapped and collapsed data matrix ready for downstream GSEA or other analysis which requires no multiple probesets per gene (Gene centric). [file 1471-2164-14-514-S4.pdf]
