# Supplementary figures and images for: Confero: an integrated contrast data and gene set platform for computational analysis and biological interpretation of omics data
Source: BMC Genomics. 2013 Jul 29;14:514. doi: 10.1186/1471-2164-14-514 (PMC3750322; doi:10.1186/1471-2164-14-514)

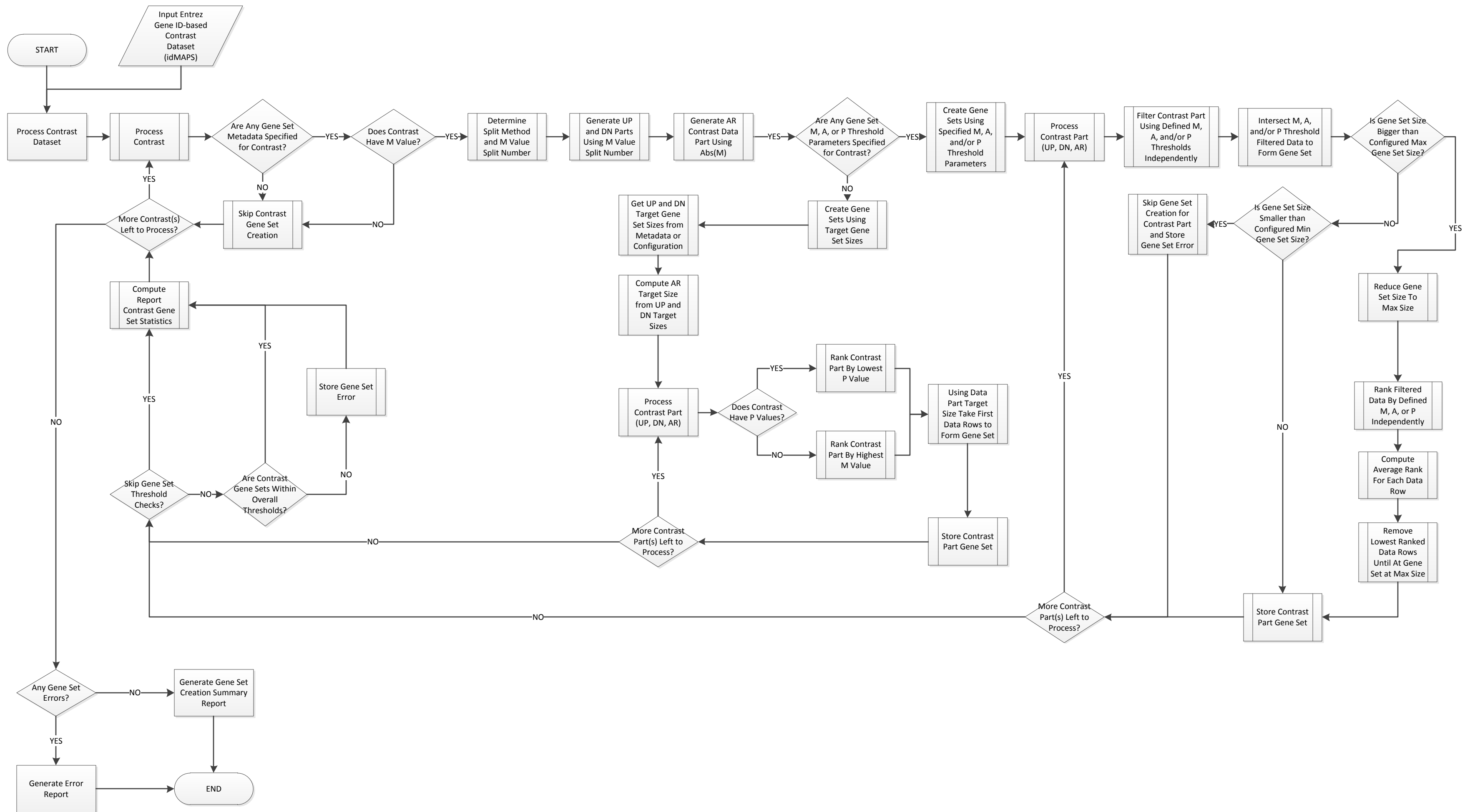

Supplement: Additional file 5: Figure S2 — Confero gene set extraction algorithm flowchart. The figure depicts the steps to extract up- (UP), down- (DN) and all- (AR) regulated genes from mapped and collapsed contrast data leading to the creation of new gene sets stored in Confero DB. [file 1471-2164-14-514-S5.pdf]
