# Supplementary material for: Confero: an integrated contrast data and gene set platform for computational analysis and biological interpretation of omics data
Source: BMC Genomics. 2013 Jul 29;14:514. doi: 10.1186/1471-2164-14-514 (PMC3750322; doi:10.1186/1471-2164-14-514)
Supplement: Additional file 10 — Confero command line interface documentation including examples. Confero can be used via Galaxy web interface or command line interface for a programmatic usage of the platform. [file 1471-2164-14-514-S10.pdf]

Confero Contrast and Gene Set Platform CLI

Environment Setup

Make sure the Perl is added to your **`$PATH`**. In your **`$HOME/.bash_profile`**:

```
PERL_HOME=/usr/local/perl/
export PERL_HOME
```

And then add the **`$PERL_HOME/bin`** to your **`$PATH`** variable declaration:

```
PATH=$PERL_HOME/bin:$PATH
export PATH
```

Confero Command Runner

The Confero command runner is:

```
cfo_run_cmd.pl --help
Usage:
  cfo_run_cmd.pl [command] [options]

  Commands:
      process_data_file
Check and process a data file (e.g. contrast data set, gene set list)

      process_submit_data_file
Check, process and submit a data file (e.g. contrast data set, gene set list)

      create_ranked_lists
Create ranked list expression profiles from a contrast dataset or contrast
file or one in Confero DB

      analyze_data
Analyze data for gene set enrichment using Confero DB, MSigDB, GeneSigDB,
etc. gene set collections

      extract_gsea_leading_edge_matrix
Extract GSEA leading edge matrix from a GSEA result

      extract_gsea_results_matrix
Extract GSEA results data matrix from one or more GSEA results

      extract_gene_set_matrix
Extract gene set matrix from specific gene sets or one or more gene set
databases

      extract_gene_set_overlap_matrix
Extract gene set overlap matrix from a gene set matrix or GSEA leading edge
matrix

      extract_contrast_data_subset
Extract a subset of contrasts from an contrast dataset file or one in Confero
DB

  Options:
      --help                             Print usage message and exit

  Run cfo_run_cmd.pl [command] --help for command options
```

Process (and Submit) Data File

Examples

To process and check outputs without doing database submission:

```
cfo_run_cmd.pl process_data_file \
--data-file=/path/to/input_data_file.txt \
--data-type=idMAPS \
--report-file=/path/to/processing_report.txt \
--processed-file=/path/to/processed_data_file.txt
```

Or for convenience:

```
cfo_process_data_file.pl \
--data-file=/path/to/input_data_file.txt \
--data-type=idMAPS \
--report-file=/path/to/processing_report.txt \
--processed-file=/path/to/processed_data_file.txt
```

To output processed file to `STDOUT` and ignore report:

```
cfo_process_data_file.pl \
--data-file=/path/to/input_data_file.txt \
--data-type=idMAPS
```

To process and submit to database:

```
cfo_run_cmd.pl process_submit_data_file \
--data-file=/path/to/input_data_file.txt \
--data-type=idMAPS \
--report-file=/path/to/processing_report.txt \
--processed-file=/path/to/processed_data_file.txt
```

Command line options

Option	Required/Optional	Default Value	Description
--data-file=/path/to/input_data_file.txt	required		path to input data file
--data-type=<one of idMAPS, idList, RankedList>	required		note: might remove this requirement in the future with new file type peek
--report-file=/path/to/processing_report.txt	optional		path to processing and submission report file
--processed-file= /path/to/processed_data_file.txt	optional	STDOUT	path to processed/mapped/collapsed output data file
--id-type=<data ID type>	optional (required if #%id_type not set in data file header)		data file ID type
--output-file= /path/to/output/processed_data_file.txt	optional		path to processed mapped and collapsed output data file
--output-as-gene-symbols	optional	false	use official gene symbols as IDs instead of Entrez Gene IDs in processed/mapped/collapsed output data file
--organism="<organism name>"	optional (required only if id_type=GeneSymbol and if #%organism not set in header)		organism name e.g. "Homo sapiens" or Homo_sapiens if you don't want to use quotes
--collapsing-method= <one of contrast_data, dataset_data, rep_source_id>	optional	contrast_data	ID data collapsing method, can also be set in %#collapsing_method metadata header to override default
--skip-threshold-checks	optional	false	skip computed gene set threshold/sanity checks
--overwrite-existing	optional	false	overwrite existing dataset, metadata, and all related gene sets in Confero DB
--debug-file=/path/to/debug.out	optional		for development purposes only; path to output debugging Confero object dump file

Create Ranked Lists

Examples

To create ranked lists for all contrasts in a dataset in the Confero DB, give the contrast dataset ID:

```
cfo_run_cmd.pl create_ranked_lists \
--data-id="BioConductor_Estrogen_PW"
```

To create ranked lists for all contrasts in a contrast dataset idMAPS file on the filesystem:

```
cfo_run_cmd.pl create_ranked_lists \
--input-file=/path/to/input/idMAPS.txt
```

To put output ranked lists in a different directory than `$PWD`:

```
cfo_run_cmd.pl create_ranked_lists \
--data-id=" BioConductor_Estrogen_PW" \
--output-dir=/path/to/output/dir
```

To create a ranked list for a single contrast in the Confero DB, give the contrast ID:

```
cfo_run_cmd.pl create_ranked_lists \
--data-id="BioConductor_Estrogen_PW[Estro48]"
```

To put output ranked list in a different directory than `$PWD`:

```
cfo_run_cmd.pl create_ranked_lists \
--data-id="BioConductor_Estrogen_PW" \
--output-dir=/path/to/output/dir
```

To choose a different name and location for the output ranked list:

```
cfo_run_cmd.pl create_ranked_lists \
--data-id="BioConductor_Estrogen_PW" \
--output-file=/path/to/output/ranked_list.rnk
```

Command line options

Option	Required/Optional	Default Value	Description
`--data-id="<Confero contrast dataset or contrast ID>"`	required either `--data-id` or `--input-file`		Confero DB contrast dataset or single contrast ID
`--input-file= /path/to/input/idMAPS.txt`	required either `--data-id` or `--input-file`		path to input contrast dataset idMAPS file
`--output-dir= /path/to/output/dir`	optional	`$PWD`	output directory path; will attempt to create directory path if doesn't exist
`--output-file= /path/to/output/ranked_list.rnk`	optional	`$PWD/<contrast ID>.rnk`	ranked list output file path
`--output-id-type=<EntrezGene or GeneSymbol>`	optional	`EntrezGene`	ranked list output ID type
`--rank-column=<S or M>`	optional	`S`	rank metric used for rank column

Analyze Data

Examples

To run a GSEA Preranked on MSigDB C2 CGP and C2 CP collections in `$PWD`:

```
cfo_run_cmd.pl analyze_data \
--input-file=/path/to/ranked_list.rnk \
--gene-set-dbs="c2.cgp,c2.cp"
```

To run analysis in a specified working directory, creating directory path if doesn't exist:

```
cfo_run_cmd.pl analyze_data \
--input-file=/path/to/ranked_list.rnk \
--gene-set-dbs="c2.cgp,c2.cp"
--working-dir=/path/to/working/dir
```

To analyze with custom gene set DB collections:

```
cfo_run_cmd.pl analyze_data \
--input-file=/path/to/ranked_list.rnk \
--gene-set-dbs="c2.cp" \
--gene-set-db-file=/path/to/custom_gene_set_db_1.gmt \
--gene-set-db-file=/path/to/custom_gene_set_db_2.gmt
```

To filter gene set DB collections gene set names for certain keywords, using boolean logic:

```
cfo_run_cmd.pl analyze_data \
--input-file=/path/to/ranked_list.rnk \
--gene-set-dbs="c2.cgp,c2.cp" \
--filter-bool-expr="TNF and NFKB"
```

Command line options

Option	Required/Optional	Default Value	Description
`--input-file=/path/to/ranked_list.rnk`	required		path to input ranked list file
`--data-type=RankedList`	optional	`RankedList`	note: will remove in the future with new data type file peek
`--analysis-algorithm=GseaPreranked`	optional	`GseaPreranked`	note: will have other analysis algorithms in the future
`--id-type=<input ID type>`	optional (required if #%id_type not set in ranked list header)		file ID type
`--organism="<organism name>"`	optional (required only for `id_type=GeneSymbol` and if `#%organism` not set in header)		organism name e.g. "Homo sapiens" or Homo_sapiens if you don't want to use quotes
`--working-dir=/path/to/working/dir`	optional	`$PWD`	path to working directory where all working and output files will go, will attempt to create directory path if doesn't exist
`--analysis-name="<analysis name>"`	optional	`basename` of input file	GSEA report analysis label
`--scoring-scheme=<scoring scheme>`	optional	`weighted`	scoring scheme, `weighted`, `weighted_p2` or `weighted_p1.5`
`--gene-set-dbs="<CSV of gene set DB IDs>"`	optional (required if `--gene-set-db-file` not set)		CSV of gene set DB collections IDs to use in analysis
`--gene-set-db-`	optional (required if		path to custom

`file=/path/to/gene/set/db/file.gmt`	`--gene-set-dbs` not set)		gene set DB collection files (in *.gmt format) to use in analysis; this option can be set **multiple** times for multiple additional gmt file databases
`--filter-bool-expr="<boolean filter expression>"`	optional		free-text filter string of gene set names in gene set DB collections to use in analysis, e.g. "TNF and NFKB"
`--do-ar-analysis`	optional	`false`	do special AR analysis with MSigDB c2.cgp and/or Confero DB AR gene set DB collection
`--debug-file=/path/to/debug.out`	optional		for development purposes only; path to output debugging Confero object dump file

There are other Confero annotation filter command line options but you need to know what is available to know what to filter by, more useful via the Galaxy GUI.

The gene set DB IDs used in `--gene-set-dbs` option are:

```
cfodb (all of Confero DB)
cfodb.contrasts
cfodb.uploads
msigdb (all of MSigDB)
c1.all
c2.all
c2.cgp
c2.cp.biocarta
c2.cp.kegg
c2.cp.reactome
c2.cp
c3.all
c3.mir
c3.tft
c4.all
c4.cgn
c4.cm
c5.all
c5.bp
c5.cc
c5.mf
genesigdb (all of GeneSigDB)
```

⚠ If you build your own gene set database *.gmt files and want to use them during GSEA with the `--gene-set-db-file` option, make sure all the gene symbols in your *.gmt file are CAPITALIZED as the Broad's GSEA implementation utilizes only capitalized gene symbols regardless of species

The typical GSEA output will look like this:

```
drwxr-x--- 3 lhermida lhermida   65536 Nov 24 15:06
dataset_677.analysis.GseaPreranked.1322143533197
-rw-r----- 1 lhermida lhermida  173366 Nov 24 15:05 dataset_677.rnk
-rw-r----- 1 lhermida lhermida 9024504 Nov 24 15:05 GENE_SYMBOL.chip
-rw-r----- 1 lhermida lhermida   20291 Nov 24 15:06 gsea.out
drwxr-x--- 2 lhermida lhermida   32768 Nov 24 15:05 nov24
```

Simply do:

```
firefox dataset_677.analysis.GseaPreranked.1322143533197/index.html
```

to open the report web site. All GSEA result data files are in that directory as well.

> Using the Confero GSEA module command line program you get all the many things Confero does to make GSEA much better. The main disadvantage of running any Confero tools via the command line and not via Galaxy is that you can't take advantage of Galaxy goodies like cluster integration, job parallelization, and workflows. A future enhancement will be to allow the Confero webservice to be run via Galaxy.

Extract GSEA Leading Edge Matrix

Examples

```
cfo_run_cmd.pl extract_gsea_leading_edge_matrix \
--gsea-results-dir=/path/to/gsea/results/dir \
--output-file=/path/to/output/matrix.txt \
--output-type=B \
--fdr-cutoff=0.5 \
--enrichment-type=all
```

Command line options

Option	Required/Optional	Default Value	Description
`--gsea-results-dir= /path/to/gsea/results/dir`	required		path to GSEA results directory
`--output-type=<B or R or M>`	required		output matrix field type B = boolean, R = rank in list, M = rank metric
`--fdr-cutoff=<between 0 and 1>`	required		gene set FDR cutoff to include in output
`--enrichment-type=<all or pos or neg>`	optional	`all`	which GSEA enrichment results to include in output
`--output-file= /path/to/output/matrix.txt`	optional	`STDOUT`	output matrix file path
`--include-annots`	optional	`false`	include gene annotation columns

Extract GSEA Results Matrix

Examples

```
cfo_run_cmd.pl extract_gsea_results_matrix \
--gsea-results-dir=/path/to/gsea/results/dir_1 \
--gsea-results-dir=/path/to/gsea/results/dir_2 \
--gsea-results-dir=/path/to/gsea/results/dir_3 \
--output-file=/path/to/output/matrix.txt
```

Command line options

Option	Required/Optional	Default Value	Description
`--gsea-results-dir=/path/to/gsea/results/dir`	required		path to GSEA results directory, usually specified **multiple** times for each related GSEA results directory to process
`--output-columns="<CSV of GSEA results column names>"`	optional	`NES,FDR Q-VAL,RANK AT MAX`	GSEA results columns to include in output; columns available: SIZE, ES, NES, NOM P-VAL, FDR Q-VAL, FWER P-VAL, RANK AT MAX, LEADING EDGE
`--output-file=/path/to/output/matrix.txt`	optional	`STDOUT`	output matrix file path

Extract Gene Set Matrix

⚠ This particular CLI command has a number of different options, much easier to do via the Confero Galaxy UI

Extract Gene Set Overlap Matrix

Examples

```
cfo_run_cmd.pl extract_gene_set_overlap_matrix \
--input-file=/path/to/input/matrix.txt \
--output-file=/path/to/output/overlap_matrix.txt \
--output-type=pct_overlap
```

Command line options

Option	Required/Optional	Default Value	Description
`--input-file=/path/to/input/matrix.txt`	required		path to input GSEA leading edge matrix or a gene set matrix
`--output-type=<num_overlap or pct_overlap`	required		output matrix fields to have either number of genes overlap or percentage overlap
`--output-file=/path/to/output/overlap_matrix.txt`	optional	`STDOUT`	path to output overlap matrix file

Extract Contrast Data Subset

Examples

To extract a contrast data subset from a dataset in Confero DB:

```
cfo_run_cmd.pl extract_contrast_data_subset \
--contrast-dataset-id="BioConductor_Estrogen_PW" \
--output-file=/path/to/output/subset_idMAPS.txt \
--contrast-name="Estro48" \
--contrast-name="Estro10"
```

or

```
cfo_run_cmd.pl extract_contrast_data_subset \
--contrast-dataset-id="BioConductor_Estrogen_PW" \
--output-file=/path/to/output/subset_idMAPS.txt \
--contrast-names="Estro48,Estro10"
```

or

```
cfo_run_cmd.pl extract_contrast_data_subset \
--contrast-dataset-id="BioConductor_Estrogen_PW" \
--output-file=/path/to/output/subset_idMAPS.txt \
--contrast-idx=1 \
--contrast-idx=4
```

or

```
cfo_run_cmd.pl extract_contrast_data_subset \
--contrast-dataset-id="BioConductor_Estrogen_PW" \
--contrast-idxs="1,4"
```

To extract a contrast data subset from a contrast dataset idMAPS file:

```
cfo_run_cmd.pl extract_contrast_data_subset \
--input-file=/path/to/input/idMAPS.txt \
--output-file=/path/to/output/subset_idMAPS.txt \
--contrast-name="TNF" \
--contrast-name="SuS"
```

To write output to `STDOUT`:

```
cfo_run_cmd.pl extract_contrast_data_subset \
--input-file=/path/to/input/idMAPS.txt \
--contrast-name="TNF" \
--contrast-name="SuS"
```

Command line options

Option	Required/Optional	Default Value	Description
`--contrast-dataset-id="<Confero DB contrast dataset ID>"`	require either `--contrast-dataset-id` or `--input-file`		Confero DB contrast dataset ID
`--input-file=/path/to/input/idMAPS.txt`	required either `--contrast-dataset-id` or `--input-file`		path to contrast dataset idMAPS file
`--contrast-name="<contrast name to extract>"`	one of `--contrast-*` options required		contrast name to extract; can be set **multiple** times one for each contrast
`--contrast-idx=<contrast idx extract>`	one of `--contrast-*` options required		contrast idx to extract; can be set **mutliple** time one for each contrast idx
`--contrast-names="<CSV of contrast names to extract>"`	one of `--contrast-*` options required		CSV list of contrast names to extract
`--contrast-idxs="<CSV of contrast idxs to extract>"`	one of `--contrast-*` options required		CSV list of contrast idxs to extract
`--output-file= /path/to/output/subset_idMAPS.txt`	optional	`STDOUT`	path to output subset contrast dataset idMAPS file
`--debug-file=/path/to/debug.out`	optional		for development purposes only; path to output debugging Confero object dump file

Confero Information

The Confero get information command:

```
cfo_get_info.pl --help
Usage:
  cfo_get_info.pl [options] [argument]

  Argument:
      array_types
      id_types
      contrast_dataset_ids
      contrast_ids
      contrast_names
      contrast_gene_set_ids
      gene_set_ids
      annotations
      organisms
      gene_set_types

  Options:
      --as-json                    Return JSON (default false)
      --as-tuples                  Return tuples (default false)
      --with-empty                 Start with an empty tuple (default false)
      --help                       Display usage message and exit
      --version                    Display program version and exit
```
