# Supplementary material for: Confero: an integrated contrast data and gene set platform for computational analysis and biological interpretation of omics data
Source: BMC Genomics. 2013 Jul 29;14:514. doi: 10.1186/1471-2164-14-514 (PMC3750322; doi:10.1186/1471-2164-14-514)
Supplement: Additional file 9 — Confero software architecture schematic diagram showing the various platform components and how they integrate with each other. Users currently interact with Confero via the web browser or command line interface (CLI). Users execute Confero tools via the Galaxy user interface or CLI commands and can view and manage data in Confero via the Confero web application. Users can leverage Galaxy functionalities with Confero tools such as workflow building and execution, parallel and asynchronous job processing, and sharing and publishing of results. Confero administration is performed using management scripts which update annotations from NCBI Entrez Gene, Affymetrix, Illumina, Agilent, and GEO and then can reprocess Confero DB data using updated annotations. [file 1471-2164-14-514-S9.pdf]

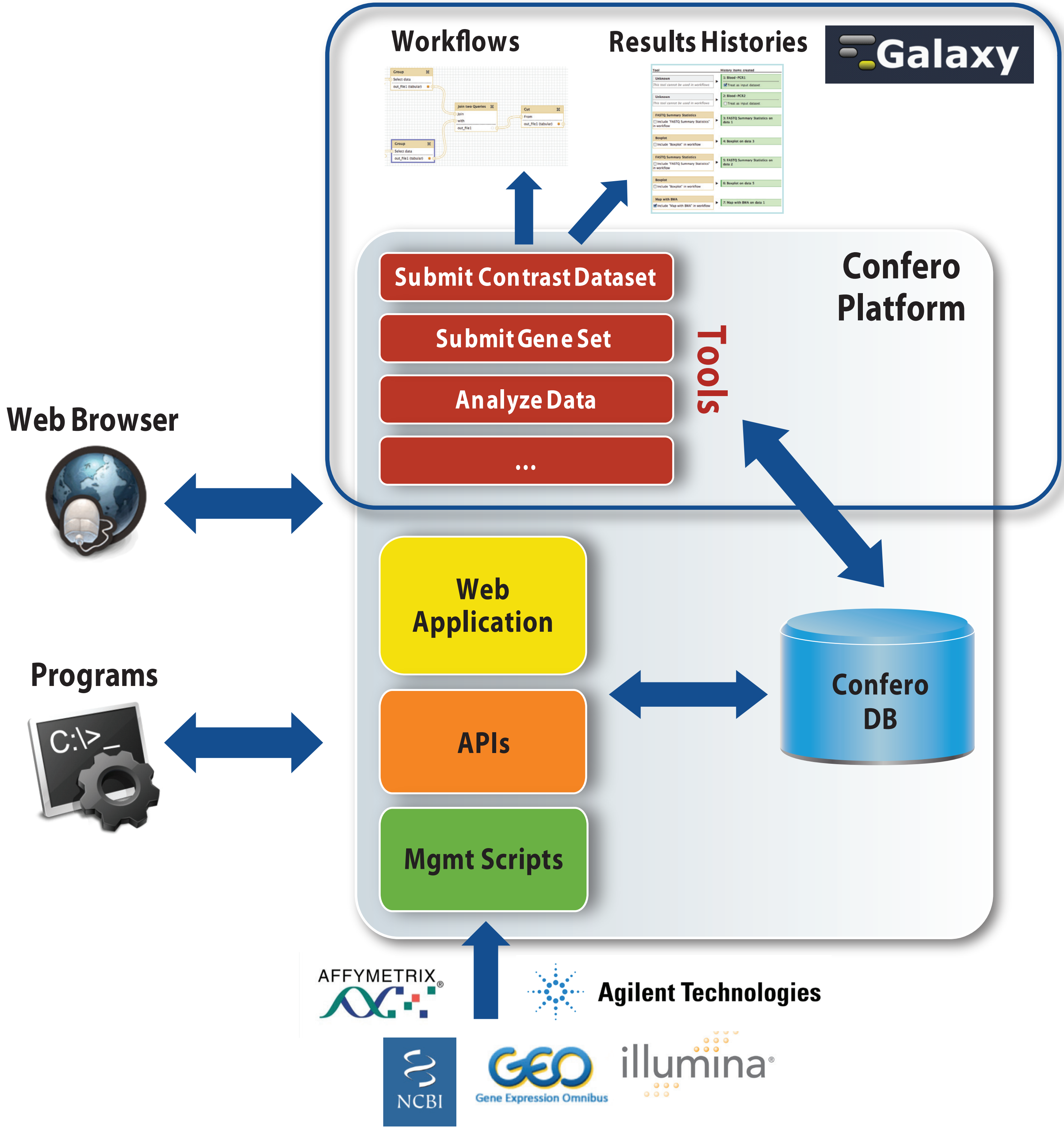
Workflows
Results Histories
Galaxy
Submit Contrast Dataset
Submit Gene Set
Analyze Data
...
Tools
Confero
Platform
Web Browser
Programs
C:\>_
Web
Application
APIs
Mgmt Scripts
Confero
DB
AFFYMETRIX
Agilent Technologies
NCBI
GEO
Gene Expression Omnibus
illumina
